# Call for emergency action to limit global temperature increases, restore biodiversity and protect health

**DOI:** 10.1136/bmjsrh-2021-201314

**Published:** 2021-09-05

**Authors:** Lukoye Atwoli, Abdullah H Baqui, Thomas Benfield, Raffaella Bosurgi, Fiona Godlee, Stephen Hancocks, Richard Horton, Laurie Laybourn-Langton, Carlos Augusto Monteiro, Ian Norman, Kirsten Patrick, Nigel Praities, Marcel GM Olde Rikkert, Eric J Rubin, Peush Sahni, Richard Smith, Nicholas J Talley, Sue Turale, Damián Vázquez

**Affiliations:** 1 East African Medical Journal, Nairobi, Kenya; 2 Journal of Health, Population and Nutrition, Baltimore, Maryland, USA; 3 Danish Medical Journal, Copenhagen, Denmark; 4 PLOS Medicine, Cambridge, UK; 5 The BMJ, London, UK; 6 British Dental Journal, London, UK; 7 The Lancet, London, UK; 8 UK Health Alliance on Climate Change, London, UK; 9 Revista de Saúde Pública, São Paulo, Brazil; 10 International Journal of Nursing Studies, London, UK; 11 CMAJ, Ottawa, Ontario, Canada; 12 Pharmaceutical Journal, London, UK; 13 Dutch Journal of Medicine, Nijmegen, The Netherlands; 14 NEJM, Boston, Massachusetts, USA; 15 National Medical Journal of India, New Delhi, India; 16 Medical Journal of Australia, Newcastle, New South Wales, Australia; 17 International Nursing Review, Geneva, Switzerland; 18 Pan American Journal of Public Health, Washington, DC, USA

**Keywords:** health services research, outcome and process assessment, outcome assessment, health care

Wealthy nations must do much more, much faster.

The United Nations General Assembly in September 2021 will bring countries together at a critical time for marshalling collective action to tackle the global environmental crisis. They will meet again at the biodiversity summit in Kunming, China, and the climate conference (Conference of the Parties (COP)26) in Glasgow, UK. Ahead of these pivotal meetings, we—the editors of health journals worldwide—call for urgent action to keep average global temperature increases below 1.5°C, halt the destruction of nature and protect health.

Health is already being harmed by global temperature increases and the destruction of the natural world, a state of affairs health professionals have been bringing attention to for decades.^1^ The science is unequivocal; a global increase of 1.5°C above the preindustrial average and the continued loss of biodiversity risk catastrophic harm to health that will be impossible to reverse.^2 3^ Despite the world’s necessary preoccupation with COVID-19, we cannot wait for the pandemic to pass to rapidly reduce emissions.

Reflecting the severity of the moment, this editorial appears in health journals across the world. We are united in recognising that only fundamental and equitable changes to societies will reverse our current trajectory.

The risks to health of increases above 1.5°C are now well established.^2^ Indeed, no temperature rise is ‘safe’. In the past 20 years, heat-related mortality among people aged over 65 has increased by more than 50%.^4^ Higher temperatures have brought increased dehydration and renal function loss, dermatological malignancies, tropical infections, adverse mental health outcomes, pregnancy complications, allergies, and cardiovascular and pulmonary morbidity and mortality.^5 6^ Harms disproportionately affect the most vulnerable, including children, older populations, ethnic minorities, poorer communities and those with underlying health problems.^2 4^

Global heating is also contributing to the decline in global yield potential for major crops, falling by 1.8%–5.6% since 1981; this, together with the effects of extreme weather and soil depletion, is hampering efforts to reduce undernutrition.^4^ Thriving ecosystems are essential to human health, and the widespread destruction of nature, including habitats and species, is eroding water and food security and increasing the chance of pandemics.^3 7 8^

The consequences of the environmental crisis fall disproportionately on those countries and communities that have contributed least to the problem and are least able to mitigate the harms. Yet no country, no matter how wealthy, can shield itself from these impacts. Allowing the consequences to fall disproportionately on the most vulnerable will breed more conflict, food insecurity, forced displacement and zoonotic disease, with severe implications for all countries and communities. As with the COVID-19 pandemic, we are globally as strong as our weakest member.

Rises above 1.5°C increase the chance of reaching tipping points in natural systems that could lock the world into an acutely unstable state. This would critically impair our ability to mitigate harms and to prevent catastrophic, runaway environmental change.^9 10^

## Global targets are not enough

Encouragingly, many governments, financial institutions and businesses are setting targets to reach net-zero emissions, including targets for 2030. The cost of renewable energy is dropping rapidly. Many countries are aiming to protect at least 30% of the world’s land and oceans by 2030.^11^

These promises are not enough. Targets are easy to set and hard to achieve. They are yet to be matched with credible short-term and longer-term plans to accelerate cleaner technologies and transform societies. Emissions reduction plans do not adequately incorporate health considerations.^12^ Concern is growing that temperature rises above 1.5°C are beginning to be seen as inevitable, or even acceptable, to powerful members of the global community.^13^ Relatedly, current strategies for reducing emissions to net zero by the middle of the century implausibly assume that the world will acquire great capabilities to remove greenhouse gases from the atmosphere.^14 15^

This insufficient action means that temperature increases are likely to be well in excess of 2°C,^16^ a catastrophic outcome for health and environmental stability. Critically, the destruction of nature does not have parity of esteem with the climate element of the crisis, and every single global target to restore biodiversity loss by 2020 was missed.^17^ This is an overall environmental crisis.^18^

Health professionals are united with environmental scientists, businesses and many others in rejecting that this outcome is inevitable. More can and must be done now—in Glasgow and Kunming—and in the immediate years that follow. We join health professionals worldwide who have already supported calls for rapid action.^1 19^

Equity must be at the centre of the global response. Contributing a fair share to the global effort means that reduction commitments must account for the cumulative, historical contribution each country has made to emissions, as well as its current emissions and capacity to respond. Wealthier countries will have to cut emissions more quickly, making reductions by 2030 beyond those currently proposed^20 21^ and reaching net-zero emissions before 2050. Similar targets and emergency action are needed for biodiversity loss and the wider destruction of the natural world.

To achieve these targets, governments must make fundamental changes to how our societies and economies are organised and how we live. The current strategy of encouraging markets to swap dirty for cleaner technologies is not enough. Governments must intervene to support the redesign of transport systems, cities, production and distribution of food, markets for financial investments, health systems, and much more. Global coordination is needed to ensure that the rush for cleaner technologies does not come at the cost of more environmental destruction and human exploitation.

Many governments met the threat of the COVID-19 pandemic with unprecedented funding. The environmental crisis demands a similar emergency response. Huge investment will be needed, beyond what is being considered or delivered anywhere in the world. But such investments will produce huge positive health and economic outcomes. These include high-quality jobs, reduced air pollution, increased physical activity, and improved housing and diet. Better air quality alone would realise health benefits that easily offset the global costs of emissions reductions.^22^

These measures will also improve the social and economic determinants of health, the poor state of which may have made populations more vulnerable to the COVID-19 pandemic.^23^ But the changes cannot be achieved through a return to damaging austerity policies or the continuation of the large inequalities of wealth and power within and between countries.

## Cooperation hinges on wealthy nations doing more

In particular, countries that have disproportionately created the environmental crisis must do more to support low-income and middle-income countries to build cleaner, healthier and more resilient societies. High-income countries must meet and go beyond their outstanding commitment to provide $100 billion a year, making up for any shortfall in 2020 and increasing contributions to and beyond 2025. Funding must be equally split between mitigation and adaptation, including improving the resilience of health systems.

Financing should be through grants rather than loans, building local capabilities and truly empowering communities, and should come alongside forgiving large debts, which constrain the agency of so many low-income countries. Additional funding must be marshalled to compensate for inevitable loss and damage caused by the consequences of the environmental crisis.

As health professionals, we must do all we can to aid the transition to a sustainable, fairer, resilient and healthier world. Alongside acting to reduce the harm from the environmental crisis, we should proactively contribute to global prevention of further damage and action on the root causes of the crisis. We must hold global leaders to account and continue to educate others about the health risks of the crisis. We must join in the work to achieve environmentally sustainable health systems before 2040, recognising that this will mean changing clinical practice. Health institutions have already divested more than $42 billion of assets from fossil fuels; others should join them.^4^

The greatest threat to global public health is the continued failure of world leaders to keep the global temperature rise below 1.5°C and to restore nature. Urgent, society-wide changes must be made and will lead to a fairer and healthier world. We, as editors of health journals, call for governments and other leaders to act, marking 2021 as the year that the world finally changes course.
